# The Hospital Nursing Supplement

**Published:** 1893-07-01

**Authors:** 


"The Hospital" Nursing Mirror.

Being the Extra Nursing Supplement of "The Hospital" Newspaper.

[Contributions for this Supplement should be addressed to the Editor, The Hospital, 428, Strand, London, W.C., and should have the word "Nursing" plainly written in left-hand top corner of the envelope.]

### News from the Nursing World.

### THE ROYAL WEDDING.

Many postcards have reached the office this week, and evidently the idea of a water picnic has been received with great favour. Numbers of the R.N.P.F. nurses seem to think they can be present if the day is fixed for after the wedding. Of course, some write regretfully that their duties will prevent their attendance at any fête, whilst others cheerfully anticipate getting a peep at the festivities on the wedding day in an independent manner, and also joining whatever plan is most generally favoured by the whole body of nurses. Our hope of finding an available space to erect a stand from which the Pension Fund Nurses could view the procession, must, we fear, be given up, as on most careful inquiry we find that prices are, alas, quite prohibitive.

### LOYALTY STILL LIVES.

People are fond in every-day conversations of adopting a carping and critical attitude towards "the powers that be," therefore it is really pleasant to find how a general holiday and a Royal procession can bring to the surface instinctive English loyalty. Jubilee Day was a magnificent instance of this, for many folks who had aired their own particular views of "how they would govern the country" were compelled by the universal rejoicing to descend unceremoniously to sympathetic communion with the cheerful and cheering masses. A general festival draws people together even more surely than a common sorrow, and the Royal wedding is an event to rouse all sleeping loyalty. The Princess of Wales is almost the only woman in England who is never subjected to criticism. As President of the R.N.P.F., she is specially dear to nurses, and they can always confidently rely upon her kindly interest. Once upon a time Miss Florence Nightingale was as universally adored, and even in these days of discussion it is only a small and weak section of the nursing world which has ventured to disparage the judgment of the Queen of Nurses because she does not agree with their particular scheme.

### WHERE ARE THE MATRONS?

One question occurs to every nurse after reading the "full text of the Charter," which we published last week. Where are the representative Matrons of the large London and provincial training schools? Why are the superintendents of these hospitals and infirmaries conspicuously absent from the appended list of ladies. Fourteen names appear, and two of them are indeed associated with the great hospital of St. Bartholemew's, for they are those of the late as well as the present Matron. The former, Mrs. Fenwick, and Miss Wood, once of Great Ormond Street, being late, not acting, superintendents of nurses, the list is reduced to twelve. Of these one is the Matron of Middlesex Hospital, which has only 307 beds, and another is at the Royal Infirmary, Bristol, where there are 264. The remaining ladies supervise hospitals of less than 200 beds each. Thus the Scotch, Irish, and large provincial nurse training schools are not represented at all, any more than such hospitals as St. Thomas's, Westminster, King's, Guy's, Charing Cross, St. Mary's, the London, or the Royal Free. We may well ask where are the Matrons? And, at any rate, the absence of their names is sufficiently significant of their unwillingness to be associated with movements which they judge detrimental to the dignity of trained nurses.

### ANGELS VERY MUCH DISGUISED.

Surely we shall have to believe in Scotch rather than Irish blarney very soon, now that a North-country physician has talked publicly of nursing as "an angelic profession," and called a princess an "archangel." No wonder Dr. Heron Watson's speech in Scotland called forth laughter as well as applause the other day. We could wish that all members of the "angelic profession" would condescend to garb themselves decorously in the streets. Modest and suitable uniforms are certainly the prettiest of all dresses, but they are very rare this season. Walking in Oxford Street we meet (alas, that it should be so!) nurses who make their professional sisters indulge in the blushes they themselves obviously "wear unseen" because of the rouge and powder which hides and disfigures every womanly grace. Rough hair and a light-hued, trailing gown with high sleeves—these last possibly shaped to disguise the budding wings—no cloak, and a great, rattling chatelaine—was this a nurse's outfit? The face of the wearer was not that of a woman in whose care a wise doctor or anxious parent would like to trust a patient seriously ill. No true nurse, respecting herself and her calling, could thus ignore the fitness of things. A trailing dress evinces lack of conscientiousness; a jingling chatelaine and high-heeled shoes show inconsiderateness for others; whilst disordered hair and crumpled veil are signs of untidiness which "he who runs may read." Nurses are more talked of and more written of than they have ever been before, but the thoughtful ones should not forget that it is their duty to try and influence their weaker sisters to be modest, quiet, and forgetful of self. A good woman, thoroughly trained, will suffice for such patients as neither admire artificial complexions nor sigh for "angelic" visitants.

### A PERMANENT HOME.

The Staffordshire Institution for Nurses, which has its head-quarters at Stoke-upon-Trent, has sent out a satisfactory twenty-first annual report. The subscriptions for the past year have almost covered the expenses of the work done gratuitously, or at reduced terms. The nurses have hitherto lived in lodgings between their cases, as there has been no Home for them, and the Committee now consider it would add greatly to the comfort of the workers if a house were provided, where also they would be cared for when sick. A legacy from a Mrs. Hitchman makes this plan feasible and doubtless the nurses will benefit considerably by the proposed arrangement.

### WISE LOCAL GOVERNMENT.

The Local Government Board have addressed a very sensible letter to the Ashton Guardians respecting the appointment made by the latter of a non-resident night nurse. How any committee could countenance such an arrangement may well puzzle people with no personal experience of Boards of Guardians. The Local Government Board have withheld their formal sanction of Alfred Turner's appointment until their letter has been replied to, and they say: "They were of opinion that it was very desirable that such officers should reside on the premises, in order that it might ensure them the proper amount of rest during the day, which was essential to enable them to discharge efficiently their duties during the night." If the responsibilities of night nursing were as clear to other Boards as to the one in question, sick paupers would fare better.

### PLAIN AND UNATTRACTIVE DUTIES.

Although a tent was furnished and ready for small-pox cases at Pershore the other day, and it was advisable to make use of it for a woman in the workhouse who was found to have this disease, no nurse could be obtained for the case. The institution at Worcester could not supply one, and four or five other associations returned the same answer. Eventually the poor woman's husband volunteered to take care of her until a nurse could be procured, and arrangements to meet the emergency were admirably carried out by Mr. Fosbroke. Good women tell us they are willing to nurse lepers, whose condition, by the way, often requires no special nursing at all, or they say they will go to cholera cases, for which adequate arrangements have been already systematically made, but small-pox and scarlet fever they avoid. These diseases are too commonplace, and so governors of isolation hospitals are driven to advertise persistently for trained workers, and to take very young probationers at good salaries, because many more competent women turn aside from present duties in search of idealised and possibly non-existent ones.

### OPPOSED PROGRESS.

The proposed improvements in the system of nursing at Cheltenham Workhouse are again threatened with postponement. Mr. Margrett has "given notice of his intention to move the rescinding of the recent resolution" to reform matters. Our readers will remember that on April 29th, under the heading "Twenty-eight Years After," we mentioned the fact that in 1865 the Central Poor-Law Authority had strongly recommended the Guardians to appoint paid nurses. However, the advice was not acted upon, and in The Hospital of May 6th we referred to the kind of paupers employed in the present year to look after the inmates of Cheltenham Workhouse Infirmary. One attendant was an imbecile, and the others suffered themselves from rheumatism, gout, and blindness, and their ages ranged from sixty-five to eighty-two years. Even at the meeting where these sad facts were discussed, the Guardians did not agree unanimously as to the need for changes. Perhaps these gentlemen are impervious to disease and sickness in their own persons, and hence the golden rule fails in application. Otherwise we might venture to suggest that when illness sets foot within their own homes they should be themselves nursed entirely by the aged paupers whom they estimate so highly. The care and skill which such persons possess could be accurately guaged and reported on by any Guardian who had conscientiously given a personal trial to attendants of the class whom he has persistently upheld in opposition to the teaching, "To do unto all men as I would they should do unto me."

### SCOTCH BRANCHES OF THE INSTITUTE.

A pleasant gathering of the Scotch Branches of the Queen Victoria Jubilee Institute Nurses took place in Edinburgh on June 20th. The President, Princess Louise, sent a congratulatory telegram to the Hon. Secretary, Miss Guthrie Wright, and the meeting was altogether an enjoyable one. Representatives from over a dozen branches in Scotland appeared at the District Training Home at Edinburgh on this annual anniversary.

### SCOTCH WORKHOUSE NURSES.

Nineteen poorhouses in Scotland possess complete nursing staffs on the scale prescribed by the Central Authority. If the moderate requirements of the Board of Supervision are complied with, "there is then repaid," writes an official to *The Scotsman*, "from the medical relief grant, one-half the salary of each trained nurse, with three shillings a-week towards her maintenance. Thus, for a nurse at £26 a-year, the Committee receive an annual grant of £20 16s."

### NEW NURSING ARRANGEMENTS IN INDIA.

It has been decided that the duties of the Indian Nursing Service shall for the future include the supervision of the station hospitals for the wives and children of British soldiers. This will necessitate a large increase of the staff of Nursing Sisters. We d onot understand what is to become of the Matrons who have hitherto been employed in the station hospitals, and who are said to be trained midwives and nurses. Are the Nursing Sisters to supersede or supervise them? In either case it will be necessary to choose Sisters who have had special experience of women's diseases as well as a thorough general training. At the South Station Hospital in the Secunderabad district four Sisters are established, and these are also held responsible for the nursing of sick officers received in the special "officers' ward" attached to the hospital.

### CHICAGO.

Miss Isabel Hampton, of Johns Hopkins Hospital, has been reading an interesting paper at the Nurses' Congress on "The Standards of Education for Nurses," and said that, in contrasting present methods with past ones, we might feel "some little complacency. But we should not be content to stand still." Miss Hampton gave an excellent summary of the qualities needed in a nurse, concluding, "The nurse must be strong, mentally and physically, and have infinite tact. . . . . She must have knowledge of men and things in many states, a wide catholicity of sympathy, a strength that springs from knowledge, and the magnanimity that springs from strength." Miss Hampton is in favour of the term of training in America being extended to three years, and she considers that before entering a hospital every woman should be instructed in housekeeping and in practical economy.

### Sanitation for Nurses.

By a Sanitary Inspector.

### XI.—SANITARY PROVISIONS IN FOREIGN COUNTRIES.

As regards many of the regulations in some of the chief European countries we are largely indebted to Dr. Palenberg for much useful and interesting information on this important branch of sanitation. For the whole of France the regulations in Paris are the most complete and are enforced with the most regularity; the other large towns generally follow the example of the capital at a distance, and as a rule, at a very long distance.

Paris is provided with two ambulance stations, one on the right bank of the Seine near the Hospital of Saint Antoine, and the other on the left bank near the Hospital for Sick Children. Each of these stations is very well provided with ambulances for the removal of patients suffering from infectious diseases, and special ambulances are used for each disease. Thus of the twelve ambulances at these stations ten are reserved exclusively for small-pox, measles, diphtheria, scarlet fever, and typhoid, whilst the remaining two carriages are kept for other diseases, such as erysipelas, whooping-cough, &c. The regulations as to the use of these ambulances are very stringent, and to prevent any chance of mistake the building in which these are kept is divided into six separate compartments. A certain number of nurses are attached to each station, who live in the adjoining hospital, and have to take it in turn to accompany the ambulance when sent to fetch a patient.

The staff at each station consists of the chief officer, two coachmen, and a servant. The ambulances are built on the same principle as English ones, but each is also provided with a drawer containing linen. After each time of use the ambulances are not specially disinfected, as is considered necessary in this country, but are merely washed with plenty of water. We should have imagined it would be advisable, however, to fumigate those ambulances which are used for general diseases, though in the case of the others the fact that certain carriages are reserved exclusively for special diseases may certainly obviate the necessity for frequent and thorough disinfection.

In Paris the public mortuaries are not used for the reception of those who have died from infectious diseases, the regulations specially forbidding such to be admitted, and bodies are only admitted "on the production of a certificate of death from the doctor who attended the patient, stating that the death was not caused by infectious disease." There appears to be no provision whatever in France against the spread of infectious disease after death—no provision for removal from a house so as to avoid the infection of the other inmates, nor for the use of suitable disinfectants in the coffin, nor for burial to take place within a specified time.

In Belgium we find the provisions far more adequate to sanitary requirements. In Brussels there are two mortuaries, whither can be removed corpses from confined houses. One of these is situated within the town, and this can only receive those who have died of a non-infectious disease. The other is in connexion with the Evère Cemetery, and is used exclusively for those who have died of infectious diseases. Removal is effected where required by the medical officer of health, and if the family give consent, and is only compulsory in times of severe epidemic, or where the medical officer of health decides that it is necessary.

The removal is carried out in litters on wheels drawn by men attached to the Burial Department, and is as far as possible done in the evening, except in cases of urgency, when removal cannot be delayed. The regulation is that burial shall take place 48 hours after death, but this time may be shortened or lengthened by special permission. In Belgium all mortuaries are under the superintendence of the Bureau d'Hygiène, and this authority is responsible for their due ventilation and disinfection, and for all sanitary arrangements connected with them.

It is curious that in Belgium such a very weak solution of carbolic acid as two to three per cent. should be considered sufficient to prevent the chance of infection spreading from a person who has died from an infectious disease, the regulations are that such a corpse shall be "wrapped in clothes dipped in a two or three per cent. solution of carbolic acid."

The Municipal Council passed in 1880 a resolution making it illegal for any patient suffering from infectious disease to be conveyed in a public conveyance. Therefore, a special carriage is stationed at the Bureau d'Hygiène, and is placed at the disposal of the public when required for the removal of such a person. To obtain its use application has to be made to the police. After each journey the carriage is thoroughly disinfected. The driver of any public conveyance is forbidden to convey any sick person whatsoever, unless provided with a medical certificate stating that he is not suffering from any infectious disease.

In Germany removal to a mortuary after death from an infectious disease is neither compulsory, nor does a special mortuary appear to be provided for the reception of such. It is specially forbidden to expose in churches the bodies of those who have died of infectious diseases, and before removal a certificate from the district doctor is required, stating that such removal will not be hurtful from a sanitary point of view.

"The body of a person who has died in his own house of infectious disease must be placed in an isolated room, and the regulations respecting disinfection observed until the funeral, which will take place within 48 hours, unless the doctor should certify that immediate burial be necessary. Persons who have assisted in laying out the body, placing it in the coffin, and all who have come in contact with the corpse in any way whatever, must submit to disinfection. Persons attending the funeral must not meet in the house where the death has taken place." Also any concourse of people at the funeral is forbidden.

"Foreigners ill of an infectious disease must be sent back with all necessary precautions if the journey can be made without endangering life, and the distance to the frontier does not exceed 35 kilometres."

A person suffering from an infectious disease may not be removed from one private house to another without leave from the police.

In Berlin, at all events, such a patient is to be removed in a special carriage provided by the police. Removal in a public conveyance is illegal. Amongst the regulations is one providing that those who die of an infectious disease shall be wrapped in cloths soaked in carbolic, and removed in a hearse to a mortuary. Even in Berlin there is, apparently, no special building for such cases exclusively, and as it expected by the authorities that all corpses shall be removed as quickly as possible to one of the mortuaries placed at the public disposal, there must be considerable risk run.

In Austria those who die of an infectious disease are to be removed to a mortuary in the cemetery, and not to the common mortuaries in the town.

Removal of a patient suffering from an infectious disease is only permitted by authority of the chief medical officer of health, and under his immediate direction.

In Sweden also no special mortuaries are provided for those who die of an infectious disease, distinct from those in common use.

In Stockholm, as at Brussels, a special carriage is kept by the city for the removal of infectious patients, lined inside with varnished wood, and disinfected after each time of use.

### Nursing in South Africa.

(By Our Special Correspondent.)

### SOMERSET HOSPITAL, CAPE TOWN.

The loveliest sunrise I had ever seen greeted us at six a.m. one Friday morning when we slowly steamed into Table Bay and had our first view of Cape Town and its surroundings. The sky, all blue and grey and golden from the sun, not yet himself visible above the mountains; the great Table Mountain, rising grand and dark straight behind the town; the moon for a short time still supreme, shining on the Bay, which was calm and still as a lake.

To the extreme right of the town we noticed a large building surrounded by trees and looking like a castle with its battlemented towers. This was Somerset Hospital, and, after a few days in Cape Town, we had the pleasure of going over this splendid institution, which has been wonderfully improved of late years.

Part of the building is given up to the resident doctor and his family, and on the ground floor are situated the nurses' dining and sitting rooms, which are very large and airy, and the latter quite luxuriously comfortable with sofa, easy chairs, piano, &c. Then there is the Sister-Matron's room and the nurses' kitchen, where all their food is kept and cooked separately and quite apart from the main building. The staff consists of sixteen nurses, and over them are two Sisters of the All Saints' Sisterhood, and a lady who has helped the Sisters for the last ten years.

There are the usual offices on this floor, store-rooms, consulting-rooms, out-patients' department, &c., also a large bath-room, with two baths and a long row of washing basins.

On the second storey are all the wards (except the children's and a private room), the operating theatre, linen cupboards, &c. By far the majority of cases are males. There are only two women's wards, one for white and one for coloured patients, medical and surgical cases being mixed. The black faces and curly heads looked very strange against the white linen.

There are lavatories and slop sinks for each ward, but no separate little kitchens, for everything is brought from the large kitchen downstairs. A large medical and a spacious surgical ward are arranged for the men, and also two smaller medical wards. The majority of cases are medical during the summer season, and until the rains begin they are mostly typhoid. After the rains a great many cases of pneumonia; during this year they have had as many as 35 fever cases in at one time, and three years ago there were 43 in at once. Just now they have between 20 and 30, 21 of whom are German sailors off a German ironclad which came into port with the men all in various stages of the disease—22 were brought in; one, a malignant case, died on the eighth day after admission, seven were still in bed, one had a relapse, two had hæmorrhage, the remaining 16 more or less convalescent, and several of them enjoying the balcony which looks on to the Bay and the shipping in the docks. Ships' masters generally pay for their men, the lowest charge made being 3s. 6d. a day.

The operating theatre was just being scrubbed, so we could only peep in, but it looked well fitted up, and lighted, like the rest of the hospital, with electric light.

A room on this floor with a single bed is kept for very bad cases, or for patients received in a moribund condition, whom it would only distress the other sick people in the larger wards to see. A huge cooling machine keeps the milk sweet, a very large quantity being used, four pints of milk and one of beef tea in the 24 hours being the general diet of the fever patients. Then we went through a large glass door into the new wing, and reached the nurses' bed-rooms, which consist of roomy cubicles, very comfortable, and boarded three parts up to the ceiling. A corridor runs round three sides, and a bath-room and lavatories are on the opposite sides. The night nurses (four in number) keep their own cubicles for dressing, &c., but retire to sleep in two rooms, apart from the rest.

Below this is the children's ward, which is the prettiest I have seen. It was given by Lady Lock, the wife of the present governor, and from her designs the walls are covered with a dado of beautifully arranged coloured pictures from magazines, &c., and above this, in the spaces between the dark panelling, are lovely pictures of every description, painted by ladies in and about Cape Town on American cloth. One was done by Lady Lock herself, and the whole effect is charming.

There are nine beds in this ward and four in a smaller supplementary ward. One dear little boy, Georgie, has been in several times. He has curvature of the spine, and was so delighted when I played with him and his "Jack-in-the-box." Two little fellows were trotting about with bare feet in the blue hospital suits, one convalescent from fever; he had been sent from St. George's Orphanage, in Cape Town, which is also under the All Saints' Sisterhood. There is a lovely little chapel where there is service five times a week, the flowers on the altar beautifully arranged by the lady who helps the Sisters, the text over the door is, "Come unto Me all ye that labour and are heavy laden."

The last room we went into is a memorial room, furnished for a private patient, with curtains on brass rods to screen off the bed, and washing stand; and the rest of the room makes a comfortable sitting-room, with handsome walnut suite, hanging wardrobe, sideboard, table, chairs, &c., and a long window opening on to the "stoep," with a delicious view of the Bay.

The Sister never takes trained nurses from England on the staff, and generally prefers colonial girls to English ones to train. Numberless applications are made to the hospital for nurses for private cases. The Sister keeps a list of those she knows for the convenience of those who need them. There is no private staff attached to the hospital, and she resolutely refuses to diminish her ward staff by sending any of her nurses out. When there is a slack time in the hospital it is utilised in giving the nurses a little well-earned rest.

Here, as everywhere else, is to be found the good nurse and the indifferent one—the woman who does her work from pure motives, whether she has taken it up as a means of livelihood or not, also the nurse who adopts it just for the fun and excitement of the thing; the failure of the latter is a mere question of time. There is a very good extract from Florence Nightingale in an elegiac article on Agnes E. Jones, the pioneer of workhouse nursing. She says in reference to nursing as a paid or unpaid art: "Probably no person ever did that well which he did solely for money. Certainly no person ever did that well which he did not work at as hard as if he did it solely for money. If by amateurs, in art and nursing, are meant those who take it up for play, it is not art at all, it is not nursing at all. You never yet made an artist by paying him well. But an artist ought to be well paid." And in thinking of nursing as "an art which requires as exclusive a devotion, as hard a preparation as any painter's or sculptor's work," we have an answer to the remark that is so often made to one, "Oh, I could never be a nurse. I have not patience enough, and I have too much sympathy to bear the sight of suffering." Do they think (or rather it is, I believe, because they do not think at all) that when probationers enter a hospital they are all ready trained to a life of patience and self-denial, What is learnt during the years of hard work and discipline? besides knowledge of "the diseases flesh is heir to," and the method of treating these? Surely patient bearing with fretful, refractory patients, and all the other trials which daily, hourly, cross our paths. An increase of that sympathy which these good people think they have too much of, but being construed into plain English probably means too much self-consciousness, enables us to become "trained nurses" in the fullest sense of the word. No true woman can become hardened to suffering, and the death of a patient, after years of experience, causes in her feelings of awe, wonderment and sadness.

"There is no sight
Of suffering but I saw it, and was set
To succour it; and all my woman's heart
Was torn with the ineffable miseries
Which love and life have worked."

### The Nurse Dolls.

SUCH a nice party of charming dolls assembled at the office last week, and the kind senders had packed them with so much care that they all arrived in beautiful condition. It was quite refreshing to see such dainty, tidy little maidens when bigger folks were looking hot and dishevelled. And again the fine needlework in the small garments proves that some nurses are unusually skilled in this essentially womanly accomplishment. Such exquisite stitches give promise of handiness in those departments of nursing where neatness and accuracy are specially called for. Therefore the additions recently made to the hospital collection of nursing uniforms are in every respect satisfactory. A really beautiful Irish visitor is dressed in the pretty uniform worn by the nurses at the Mater Misercordiæ Hospital. Every garment is perfect and no detail forgotten, and she brought with her a delightful little bonnet of fine straw trimmed with velvet, a cloak, with hood and collar, the skirt gored and finished in a fashion calculated to make a tailor envious of the fit which the Irish ladies have succeeded in securing. From Wirrall Children's Hospital exquisitely dressed dolls have come to join our collection, and Merthyr Hospital contributed two admirably outfitted specimens. These were not only packed with great care, but labelled in a most commendably business-like fashion. The dolls from the Tunbridge Wells Hospital represent the dress of matron, sister and nurse, and are perfectly finished. The garments are in all cases so beautifully made that the little nurses appear quite ready for immediate work. One or two dark blue sisters' dresses are of materials which wash, and these are as pretty as they are hygienic. The Gloucester Children's Hospital is represented by a beautiful doll on a stand, quite a picture of a nurse. The Royal Free Hospital uniform is shown by three pretty dolls, very nicely dressed in the uniforms of the several grades of nurses. Birmingham City Asylum and Cold Ash Children's Hospital send very completely equipped dolls, and the Lancaster and Rotunda Hospitals are excellently represented. Our contributors have in each case worked conscientiously, for they have made the inside garments quite as daintily complete as the outside ones, petticoats being as neat as aprons, and the stockings and shoes delightful. Indeed, some of the stockings are such wonderful examples of fine knitting that we begin to think the fairies must have been requisitioned for the service of the little nurse dolls. When the collection returns from its present country tour, we will notify the fact to our readers, and invite them to judge for themselves at the office on the merits of the various uniforms. We must not forget the handsome specimen of an asylum nurse which has been sent by the Berry Wood staff, accurately dressed in the uniform worn at that institution, the garments being excellently fashioned and finished. Two nice little dolls have arrived from Moor Edge Children's Hospital, in the pretty costumes worn by the Sisters and Nurses there.

### The Charter.

WE call the attention of our readers to *Nursing Notes* for July, which will contain interesting matter respecting the British Nurses' Association and their Charter.

### The Royal British Nurses' Association.

WE give two letters below which appeared in the *Morning Post* of June 26th, and we think that all nurses will be considerably reassured by finding how very little power Princess Christian herself claims for the Charter :—

I have asked and obtained permission from Her Royal Highness Princess Christian of Schleswig-Holstein to make use of the following extract from a private letter I have received from Her Royal Highness, which I venture to think will remove in a great measure, if not altogether, the erroneous misapprehension which exists as to the intentions of the above Association, of which Her Royal Highness is President, and for which Her Majesty has been graciously pleased, on the recommendation of her Privy Council, to grant a Royal Charter.—Yours, &c.,

JAMES GILDEA, Colonel.

The Royal British Nurses' Association neither trains, certificates, nor employs nurses. It deals with them as individuals only without reference to their place of training, except in so far as it applies to those institutions for testimony as to efficiency, personal fitness, and good character. Having done so much it makes a record of the training of the nurses inscribed on its register or list, for the convenience and protection of the public and the medical profession. Its procedure is founded, as nearly as circumstances permit, on that of the General Council of the Medical Registration, and, such being the case, it can no more interfere with the constitution and internal arrangements of those training and nurse-employing institutions than the Council of Medical Registration interferes with the degree-bestowing Universities and the various colleges of physicians and surgeons. Neither does the Association, in so working, aim at affiliation, or in any way superseding existing nurse-training and nurse-employing institutions; on the contrary, any such action would be altogether beyond the sphere of its actions and unwarranted by the powers about to be conferred, or rather powers conferred, by the Royal Charter. Of the twelve nurse-training hospitals in London either the matron or one or more medical officers of the following institutions are members of the General Council of the Royal British Nurses' Association—viz., St. Bartholomew's, Guy's, University College, St. Mary's (Paddington), Middlesex, Charing Cross, King's College, the Royal Free, St. George's, Kensington Infirmary, Victoria Hospital for Children, Great Ormond Street Hospital, and Chelsea Infirmary. The officials of the leading hospitals all over the British Empire are also members of the Association. Forgive me for writing you all this, but I wanted to show you how really unfounded, if I may use the term, are the fears and the opposition to my Association. It interferes with no one, or with any institution. It is a large body formed for the mutual protection and help of nurses, as well as for the protection of the public and the assistance of the medical profession. I have written at some length, as I thought the information might be of use to you, and might help you to contradict any false impressions as to the work and aim of the Association. It has ever been my wish from the first to work in harmony with all those interested in the training of nurses; but I have every reason to believe that much of the opposition which I have encountered has been based on misapprehension, which I trust will now be dispelled.

### Appointments.

[It is requested that successful candidates will send a copy of their applications and testimonials, with date of election, to THE EDITOR, The Lodge, Porchester Square, W.]

CORBETT HOSPITAL, STOURBRIDGE.—Miss Jane E. Heath has been appointed Matron of the Corbett Hospital. She was trained at St. Mary's Hospital, and by the Metropolitan and National Nursing Association. Miss Heath was Superintendent of the Queen's Nurses' Institute at Cardiff, and we wish her every success in her new work.

LONDON COUNTY ASYLUM, CLAYBURY.—Miss F. E. Browne has been appointed Matron of this asylum. She was trained at Dorset Asylum, and afterwards held the post of head nurse at Menston Asylum, Leeds.

### India.

### MAHABLESHWAR—DRAWBACKS TO A HILL STATION.

The proceeds of a theatrical performance and a concert, both given by amateurs, at Mahableshwar, have been handed over to the Nursing Sisters at the Sassoon Hospital, Poona. A sojourn "among the hills" means health and refreshment for many Anglo-Indians, but this will not long remain so, according to the following paragraph which we quote from the *Bombay Gazette*: "The number of chiefs and princes who now throng the Hill give large sums to the poor and sick, while other wealthy gentlemen daily feed numbers of them. One is glad they should thus be fed and cared for, but if this goes on yearly, Mahableshwar must cease to be a sanitarium. The village itself is far too small to hold such a number of infirm, and those stricken with loathsome diseases as one meets on the road, and the only solution as to whence they come must be found in the fact that they congregate from the surrounding country for the good things they can get. Certain parts of the Hill are quite unpleasant owing to the crowds met with, some without hands, others whose feet have gone, and when one observes that these and all the ill-clothed and unwashed children from the Bazaar mix with the European children round the band-stand, one wonders whether it will long be advisable to send the little ones up to a place where they are subjected to the contagion of measles, ophthalmia, and diseases even worse than these." It is, indeed, hard lines for the white babies, who have so many trials of climate to battle through in India, that such a valuable health resort as Mahableshwar should be thus converted into a field for the spread of contagion and infection.

### More Girl Nurses.

We have repeatedly called attention to the evil practice of admitting young and immature women to infirmaries and incurable homes. Very few general hospitals now take probationers who are less than twenty-five years of age, and this is an excellent rule to follow. Some provincial institutions admit girls of twenty or twenty-two, but there are few places which emulate the Isle of Wight Infirmary in considering seventeen or eighteen a suitable age for nurses. At a recent meeting of the Board of Guardians Mr. Fellows, C.C., had the courage to ask a few questions, and to declare the present system there as unsatisfactory. Instead of inexperienced and weakly girls being engaged for the infirmary, he contended that it was quite time that competent women only should be employed. Of course, someone at once remarked that Mr. Fellows "was a young guardian," and ignorant of the difficulty of getting nurses of any description to go into the wards.

We venture to state that as soon as efficient training is provided in the infirmary, and competent and experienced nurses secured to instruct their probationers, the difficulties of getting the latter will vanish.

We congratulate Mr. Fellows on being young enough to see clearly, and on having the courage to call attention to the impropriety of girls of seventeen and eighteen being employed in the male wards.

The Isle of Wight Guardians had better put themselves into communication with the Workhouse Nursing Association and find out what is considered the best method of fitting women to hold the important and responsible position of infirmary nurses. Surely our poorer brethren merit a little better attention than that which is obtainable from the absolutely untrained young persons to whose inexperienced hands they are now trusted at the Isle of Wight Infirmary.

### Everybody's Opinion.

*[Correspondence on all subjects is invited, but we cannot in any way be responsible for the opinions expressed by our correspondents. No communications can be entertained if the name and address of the correspondent is not given, or unless one side of the paper only be written on.]*

### A HOLIDAY HOME WANTED.

"B. M. D." writes: Do you know of any holiday home or place in Cromer where a hospital nurse could spend a quiet week? If you would let me know I should be much obliged to you. My holidays will come about the end of August, and I am anxious to have one week by the sea, and I thought I should prefer Cromer.

### A USEFUL SUGGESTION.

"M. F." writes: I read with deep regret of the sad accident which occurred at Bengeo College last week. Can nothing be done to prevent a recurrence of such an error? An idea has formed itself in my mind which, if made common, could surely be practically carried out by some one more competent than myself. Could not the stoppers of poison bottles be so constructed that the contents would not be easily accessible? Why not have the stoppers made to screw into the necks of the bottle's. And in cases where it is necessary to measure by drops, could not a small groove be made in the screw of the bottles neck. Then by the time a "sleepy nurse" had taken out the stopper she would be fully awake to the danger.

### Lady Roberts' Nursing Fund.

We constantly receive inquiries as to Lady Roberts' nurses, and we trust the following extract from a letter with which we have been recently favoured will answer many questions satisfactorily. Lady Roberts writes that her particular work is entirely private, but "it is so far recognised by Government that the hospitals are under the charge of the depot surgeons, and medicines are supplied from the Government dispensaries free of cost. Also soldier orderlies are supplied to assist my nurses in the officers' hospitals. I have purposely abstained from having any place of reference for nurses as I select all my own nurses myself from personal knowledge, which ensures the success of the work, and I could never undertake to answer applications from nurses."

### Trained Nurses in Workhouses.

### ST. MARY ABBOTTS' INFIRMARY.

At a meeting of the St. Mary Abbotts', Kensington, Board of Guardians on Thursday, 15th June, ten probationer nurses, who had completed their three years' term of training, received certificates from the chairman. The examiner, Mr. Walter Tyrrell, reported that all the probationers had passed a very satisfactory examination, and in the course of his remarks said, "The Kensington Infirmary must take a high place among the training schools for nurses, the knowledge of the pupils being above the average, and great pains must have been taken to render them so efficient."

Lectures are given by the Medical Superintendent, the Matron, and Assistant Medical Officer. The names of the nurses who received certificates were: Frances A. Albutt, Martha Hill, Agnes Carson, Maude Weddall, Kate Jüng, Alice Kenyon, Elizabeth Dixon, Florence Perkins, Fanny MacMahon, and Mary Brentnall.

### Where to Go.

The concluding lecture of the session will be given at the Midwives' Institute and Trained Nurses' Club on Friday evening, June 30th, at 7.45, by Mrs. Scharlieb, M.D., B.Sc. Subject: "Hæmorrhage before Parturition." Non-members' tickets 6d. each.

### Precautions Against Cholera.

NOTES BY A NURSE.

A VERY interesting lecture was given by Dr. Thorne-Thorne on June 24th, at Grosvenor House, on "Cholera: Its Prospects and Prevention." He first enumerated the times England had suffered from an epidemic of this disease, pointing out the decreased mortality at each visit, owing, doubtless, to improved sanitation. With regard to Hamburg, its great interest for us consisted in the constant intercourse between it and this country. Its ships reaching us in fan-like fashion, bound not to one or two, but to nearly every port in Great Britain. Last year Hamburg suffered the heaviest visitation of cholera it had ever experienced, and the origin of it has been traced to the polluted state of the water supply. Hamburg takes its water from the Elbe, below the emigrant Jews' quarters, the drainage from their huts flowing into the river, the water of which is supplied directly to the houses. Altona, a town that adjoins Hamburg, also receives its water from the Elbe, but it is first stored in large filter beds, and then distributed to the houses of Altona, the consequence being that during the great epidemic Altona escaped, with the exception of a few isolated cases. On visiting Hamburg a few weeks ago, Dr. Thorne-Thorne said he found tremendous efforts were being made to remedy this state of things. Immense waterworks were being built outside the town, with properly constructed filter beds, and so earnest were the authorities about this that the work was being carried on by means of the electric light by night as well as day.

As regarded the disease itself, the lecturer thought that to be properly prepared for its visits its nature must be somewhat understood. Given three conditions their combination meant cholera. These three conditions were—1st, the cholera microbe; 2nd, the condition of soil; 3rd, the predisposition of the individual.

Cholera microbes will grow on moist linen, moist and porous soil, in milk, stale foods, &c., but it hates sours and acids, and dies under a high temperature; therefore milk and all water used for drinking purposes should be boiled.

As regards local condition of soil, a moist porous soil is always liable to produce the germs of cholera. The microbe, loving oxygen, will leave its home in the moist earth, and, rushing up through the porous soil to the outer air is wafted about till it finds the third condition in the individual predisposed for its poison.

He considered that England escaped an epidemic of cholera during the late great heat through the long-continued drought, as there are still towns and villages in England where the water is supplied straight from rivers. He spoke of a village in Essex where the local condition of soil was favourable for the production of cholera, namely, first a stratum of clay, which formed a basin to collect the water, above which was a stratum of gravel, giving the moist, porous soil spoken of. The method of digging two holes in the ground adjoining a house, one to collect the water, the other to receive the drainage, still obtained favour in some places. This cesspool can only be emptied by the sanitary authorities, but as they are swayed by British public opinion, until the British public understands and realises its danger it will never trouble to acquiesce in and submit to the carrying out in its smallest details this most important work of sanitation. Therefore the County Council is now endeavouring to educate the people by means of lectures, &c., and teach them the danger they are at present exposed to.

All water and milk should be boiled before being used for food. The only objection to drinking boiled water was its flat taste, which could be easily remedied by re-aërating it by passing it through a very simple filter made of a common flower-pot, containing first a layer of coarse gravel, then one of finer gravel, and finally one of sand. Dr. Thorne-Thorne concluded his lecture by summing up the best precautions against cholera, viz., abstinance from unwholesome food and alcoholic drink, and absolute cleanliness of person, habitation, and surroundings. Amongst those present were: Princess Christian, Dr. Heron, Sir R. Rollinson, Mr. J. G. Thrupp, Sir Douglas and Lady Galton, Lady Priestley, and the Duchess of Bedford.

### For Reading to the Sick.

### RICHES.

MOST people think a great deal of riches. To be rich, to provide well for one's family, to have the pleasures and good things which accompany riches, and the attention and flattery of those who would share our wealth, is pleasant to many minds; some even heap up riches for the sake of the gold itself.

God, who is the Maker and Ruler of all material things, does not speak to us of this kind of wealth. Though the world is full of vast treasures, of gold and silver and precious stones, and its surface is covered with magnificent buildings, works of art, rich clothes, and other desirable things too numerous to be counted, yet all these perish in the using, and will one day pass quite away. The only riches that will remain will be those of God's mercy and grace. They will never perish, but will always be the same—treasures which can never grow less. No moth or rust will corrupt them; no thief can break through and steal; no use, however great, will diminish them. The Apostle Paul speaks of God as "rich in mercy," and the Psalmist says, "the Lord is good to all men, and His mercy is over all His works." Noah in the ark, Joseph in prison, Israel in Egypt, the widow in Nain, the little children Christ took in His arms to bless, the thief on the cross, the fatherless and the widow, the afflicted, the forsaken and the backslider, have all received the mercy of God, until, as the Psalmist says, the earth is full of His mercy. And yet we abuse His kindness and take advantage of it, and live in our health and strength all to ourselves, until sickness overtakes us, and then we give to God our useless, worn-out lives.

But God's mercy is infinite, and like as a father pitieth his own children, so the Lord pitieth them that fear Him.

As the human mind naturally thinks well of riches, let our minds dwell on Him who is rich unto all that call upon Him, rich in wisdom, rich in glory, rich in goodness, rich in grace. He is so generous that He will give us abundantly of all these good things if we ask Him with all our hearts. By His tender mercy He made the day-spring from on High to visit us, and brought light into a dark world; now let the heart of men be never so dark, be they steeped in misery or sin, the same Light is always at hand to shine upon them and lead to the true riches. And what comfort and satisfaction and rest there is in feeling that this is true; that we are not dependent on ourselves only, but in troubles and sickness have one mightier than ourselves who is always ready, lovingly to help us. Are we in pain, a cry for help will bring ease or patience to bear what is sent us; are we in need, a petition to Him who feeds the sparrows, and lets not one fall to the ground without His knowledge—a prayer to Him who clothes the lilies and the grass of the field in all their beauty will never be disregarded. "Are not ye much better than they?" said our dear Lord. Yes, we are His own dear children, to whom He sends all things richly that we may enjoy them. It will be our own fault if we lose our share by failing to seek for them. We have been told, "Ask and ye shall receive," *i.e.*, all things that be necessary both for our souls and bodies; "seek and ye shall find" happiness in this world and in the world to come; "knock," and the gates of the kingdom of heaven shall be opened and its treasures given to all who love the Lord Jesus in spirit and in truth.

### The Muses' Looking Glass.

### THE REPUTATION OF GEORGE SAXON.*

This little volume is as unpresuming as its contents are interesting, modern, and suggestive. When the tale is told it leaves one something to think about. One argues with oneself as to whether one would have done likewise under similar conditions. I have read no story since "Mrs. Keith's Crime," which leaves one such a hard problem to solve as "The troubles of Johann Eckert." Rudolf and Johann Eckert are brothers, natives of Southern Germany, Rudolf the counterpart of his father, Johann of his mother. "All people trusted Rudolf. Johann was not alone in thinking that something would be done when the big steadfast man made up his mind to do it. All inexperienced as he was, Johann had no fear and misgiving with Rudolf. Without him he would have felt as his old mother had done when her husband died, irresolute and fearful. That was why the vineyard had to be sold; that was why Johann had come to join his brother in Africa. Yet during her husband's lifetime Frau Eckert had been reckoned clever and strong; whilst after all it was with a strength she drew from her husband. And Rudolf and his father were as like as two oxen at night-time." But in South Africa Rudolph has only just succeeded in making both ends meet. Now comes his brother Johann, who hopes to find Rudolf prosperous and able to put him in the same road, that he may send soon for the little Gretchen who has promised to be his wife. But, alas! in that desolate spot there is no opening for Johann, and Rudolf's heart bleeds at the hopeless prospects of his brother and Gretchen. Hour after hour he ponders, and finally decides that the only chance for them is to load a waggon with goods to exchange for feathers and ivory, and to start for a two years' trading expedition into the interior leaving Rudolf's wife to manage the farm. When the two years are just about over, the brothers are making their way home as quickly as their heavily-freighted waggon and diminished team of oxen will allow. Their trading has been most successful. The thought that they are nearing home brings unlimited joy to the brothers, Johann can now send for his Gretchen, and Rudolf thinks tenderly how the wealth he is bringing shall compensate the wife he adores for her desperate struggle with poverty during his absence. There is only one cloud. About a year before, Rudolf had shot down a gemsbok, and thinking it dead went forward to secure it, but the creature in its death agony manages to rise and pierce him in the side. The pathetic account of Rudolf's lingering is too long to quote. His one desire is to live to clasp his wife once more, but the trying climate, rough fare, and jolting waggon aggravate his suffering, and he dies. With his last breath he solemnly charges his brother to let nothing prevent him from getting the waggon safe to the frontier, so that the wife and children may be secure from want for the rest of their lives. Johann, broken-hearted, buries his brother in the sandy veldt, and starts on alone to fulfil his pledge. He is nearly helpless with grief, and at every moment hesitates, for he is accustomed only to act under the direction of his brother. After three days of solitary trekking through the dessert he is thankful to meet with a group of Hottentots. They greet him amicably. Then they beg. "Baas, we're hungry; give us something to eat." Johann gives them generously of his salt beef and flour. They come presently and beg more salt. Johann finds the bag nearly empty, and hunts for another. He finds one. The contents look like salt, but putting a crystal against his tongue he finds it tastes bitter. He looks for a label, and discovers that he has got hold of the arsenic used for skin preserving. Johann trembled to think what he had nearly done, and turning to the Hottentot who was watching him, says that he has no salt. The fellow does not believe him and goes off grumbling. The ensuing days are most thrillingly told. The Hottentots, knowing he is in their power, one against so many, follow him to each encampment. When they have eaten his stores, they beg his oxen. It is useless for him to refuse. "Better give us an ox, Baas; then we go away." He spoke so insolently that Johann felt bewildered and helpless. "You have said that every time," he replied. "This time we go, Baas," said the man, with a horrible chuckle which belied his promise. And so on, day after day. With his ever-diminishing team Johann can hardly get the waggon to move at all; he knows that if he continues to sacrifice his oxen his journey must perforce end here in the desert, and all the cargo be wasted. No hope of claiming Gretchen then, Rudolf's family will starve, and Johann's oath to his dying brother will be broken. Why, oh! why is that brother not at hand to counsel him? Nothing like this would have seemed possible had he been still in command. Another night comes. He tries to think. Should he try to shoot them when they come up with him again? Alas! he is no shot, and would probably not hit one of them, whereas their spears would soon end his trouble. Then comes his great temptation. His hand falls on the supposed bag of salt. This is the strong situation of the story. Here the author reaches a very high artistic level. We hold our breath as our eyes rush along the lines. If he spare the blacks they will destroy him. If he could shoot them, every one would consider him justified, but he is unable to shoot. Must those innocent children and brave, unselfish women perish from want, and without ever hearing of Rudolf s fate or his own? Shall they be sacrificed because a man is too squeamish to destroy these animals (for they more resemble beasts than men), like the vermin that they are? Once more, "Baas, we're hungry. Meat, meat, Baas." Johann cannot speak, but points in silence. The gesture was enough for those who would have taken no refusal, and in a few minutes the fattest ox lies dying by the pool. Still Johann hesitates. "His face is white, blood runs from the lip he has bitten through." He takes a handful of the crystals. He throws them down again. One moment the deed seems easy, the next moment hard; easy—hard. He tries to pray. He cannot. He implores for a sign. Then he hears, "Baas, won't you give us some salt?" Without stopping again to think, he strides to their fire and drops into their cooking pot two handful of the shining crystals, then rushes off into the veldt that he may not witness that horrible feast. For a time he keeps his bearings by the far off glow of the camp fire, then, with a start, he realises the horror of his deed, he is determined to rush back and warn them not to eat. He has lost his way, but he rushes on and on, but in vain. At last he gets nearer, then, "Are those screams part of a Bacchanalian orgey; is it a dance of joy he sees! No, no! He puts his fingers in his ears and rushes off again into the desert, for he was too late."

To Rudolf's wife he says: "He dreamed of you the night he died, and waking, he called your name. Without him I did badly. Through that I lost so many of the oxen; they died in the desert." He never told her how, nor has he told Gretchen yet, though he means to when he has made up his mind as to whether he did right. He has not yet settled that question. The story is so well told in its short space of thirty pages that it is next to impossible to condense it further without sacrificing its pathos, and we must refer our readers to the book itself to obtain the benefit of the graphic interest the writer has infused into the story.

* "The Reputation of George Saxon, and other Stories," by Morley Roberts. (Cassell and Co.)

### Wants and Workers.

Can anyone give information as to an institution which would supply a nurse at a reduced fee to a poor person? The clergyman would have to bear all expenses. Information to be obtained of *Nurse Elliott, 5 Clevedon Place, Kemp Town, Brighton.*

### The Book World for Women and Nurses.

[We invite Correspondence, Criticism, Enquiries, and Notes on Books likely to interest Women and Nurses. Address, Editor, THE HOSPITAL (Nurses' Book World), 428, Strand, W.C.]

HEALTH GOSSIPS FOR WOMEN. By G. A. HAWKINS-AMBLER, F.R.C.S.E. (Simpkin, Marshall, and Co., London.)

This is really an excellent little book, and one that might well be in the hands of every woman, and especially in the hands of mothers. It is written essentially to meet the requirements of the present day. The headings of the chapters reveal the breadth of the subject. By concise method, each division of the subject is dealt with briefly, but as thoroughly as it is possible in a small and popular work of the kind. The book is full of common sense, and is easy and interesting in style. The author only claims that his chapters are gossips, but gossips from the pen of a Fellow of the Royal College of Surgeons and member of ever so many learned societies besides, are not to be passed over and forgotten as the term might imply. Mr. Ambler dwells on the excellence of fresh air, water, and exercise of a healthy kind. He advocates the bicycle, but is not kind to the habit of prolonged shoppings in lieu of more healthy occupation. Some women will wax indignant at any slur on their shopping expeditions. Many a woman, unconfessedly though it be, simply takes to shopping *pour passer le temps*, and we remember being asked by one of the fair sex to let her go and pay the quarterly bills, as it gave her "something to do." Diet, clothing, amusement, and the care of children, none of these are forgotten in this little "gossip" which affords so many excellent hints that we hope it may become as popular as it deserves.

THE JAPS AT HOME. By DOUGLAS SLADEN. (Hutchinson and Co.)

No doubt Japan is a country of many attractions, for Europeans who go there always come back in a state of enthusiasm and regret. Yet it would appear from Mr. Sladen's amusing volume of impressions that there are drawbacks even to life in Japan, at any rate, for the Japanese woman. "At present the greatest duchess or marchioness in the land is her husband's drudge. She fetches and carries for him, bows down humbly in the hall when my lord sallies forth on his walks abroad, waits upon him at meals, may be divorced at his good pleasure." . . . "Marriage must seem a hazardous experiment to the Japanese lady. If her husband turns out to be an adventurer she mustn't utter a word of complaint, but put it down to the credit of heaven; and if she is divorced, 'shame shall cover her till her latest hour'"—a little hard considering the very elastic character of the Seven Reasons for Divorce, which include such natural little outbreaks on the female part as (1) "disobedience to her father-in-law and mother-in-law; (4) jealousy; and (6) disturbing the harmony of kinmsen, and bringing trouble on her household by telling overmuch and prattling disrespectfully." The influence of Europeans is slowly making itself felt in the estimation and treatment of women, and the increasing tendency to adopt European costume which has been noticed among ladies of the upper classes and deplored, is doubtless

### THE BOOK WORLD FOR WOMEN AND NURSES—*continued.*

due to the new respect which it calls forth from their mankind. "The same fellow who struts into a room before his wife when she is dressed *à la Japonaise*, lets her go in first when she in dressed *à l'Européenne.*" Some of the directions for the behaviour of women extracted from the Daigaku Onna (Greater Learning for Women) illustrate better than any description the status of women in Japan. "The great life-long duty of a woman is obedience. . . . When the husband issues his instructions the wife must never disobey them. If ever her husband should inquire of her she should answer to the point—to answer in a careless fashion would be a mark of rudeness. Should her husband at any time be roused to anger, she must obey him with fear and trembling, and not set herself up against him in anger and disputatiousness. A woman should look upon her husband as if he were heaven itself, and never weary of thinking how she may yield to her husband, and thus escape Celestial castigation. Of tea and wine she must not drink over much. To temples and other like places where there is a great concourse of people she should go but sparingly till she has reached the age of forty. . . Neither should she be constantly occupied in praying. If only she satisfactorily perform her duties as a human being, she may let prayer alone without ceasing to enjoy the divine protection. Her personal adornments and the colour and pattern of her garments should be unobtrusive. It suffices for her to be neat and cleanly in her person and in her wearing apparel. It is wrong in her by an excess of care to obtrude herself on other people's notice." We quote in conclusion what Mr. Sladen calls the climax of woman according to man. "Such is the stupidity of her character that it is incumbent on her, in every particular ,to distrust herself and obey her husband." It would be instructive to have as a pendant the secret views of the Japanese women on men.

### PUBLICATIONS OF THE MONTH.

The Leisure Hour for July contains some very interesting articles. "Quiet Corners in Oxford" is devoted on this occasion to the description of college kitchens, features which do not fail to interest visitors to the University. There is an interesting and instructive article on Gout, and the little paragraph on the "Character of Gait" runs as follows: "Gait is an important part of physical expression. By his gait a man tells us whether he is fresh or tired, strong or feeble, in good health or in bad. To some extent also gait denotes occupation. The upright and somewhat rigid walk of the soldier differs largely from the rather rolling gait of the sailor; and different from both of these is the slow, jolting gait of the country labourer, which, however, is partly accounted for by his clumsy and heavy boots. In the peculiarities of gait, again, an attentive eye discovers many moral qualities. 'Slow steps, whether long or short, suggest a gentle or reflective state of mind, as the case may be; while, on the contrary, quick steps seem to speak of agitation and energy. Reflection is revealed in frequent pauses, and walking to and fro, backward and forward, the direction of the steps wavering and following every changing impulse of the mind, inevitably betrays uncertainty, hesitation, and indecision.' It might ask too curious a knowledge to distinguish by their respective gaits the miser, the spendthrift, and the philanthropist; but the proud man is almost always known by his step, the vain man to some extent, and the obstinate man not a little."

The Girl's Own Paper and the Boy's Own Paper have separate summer numbers, got up in a most attractive manner, and will form acceptable presents for juvenile friends.

